# ORAL CONCURRENT SESSION 5: Hypertension

**DOI:** 10.1002/pmf2.12013

**Published:** 2025-01-05

Abstracts 57 – 66

FRIDAY

January 31, 2025

1:30 PM – 4:00 PM

Aurora Ballroom B

MODERATORS

Hyagriv Simhan, MD, MS

Alan T. Tita, MD, PhD

